# De-escalation of axillary surgery in clinically node-negative (cN0) breast cancer: current evidence, advances, and controversies

**DOI:** 10.3389/fonc.2026.1971201

**Published:** 2026-10-12

**Authors:** Jinxiu Ma, Meizi Cui, Dongqing Li, Sijia Wang, Jian Sun, Hao Guo, Yingying Ding, Zhimin Fan, Xiaozhen Wang

**Affiliations:** 1 Department of Breast Surgery, General Surgery Center, The First Hospital of Jilin University, Changchun, Jilin, China; 2 Department of General Practice, The First Hospital of Jilin University, Changchun, China; 3 The Second Department of Gynecologic Oncology, Jilin Cancer Hospital, Changchun, Jilin, China; 4 Department of Burn and Plastic Surgery, Jilin Provincial People’s Hospital, Changchun, Jilin, China

**Keywords:** axillary de-escalation therapy, breast cancer, cN0 stage, metastatic burden, molecular subtype

## Abstract

Clinically node-negative (cN0) breast cancer accounts for a large proportion of newly diagnosed cases, and its axillary management is undergoing a paradigm shift toward de-escalation aimed at minimizing surgical morbidity. Sentinel lymph node biopsy (SLNB) has replaced axillary lymph node dissection (ALND) for nodal staging in cN0 disease, and subsequent trials have questioned the need for completion ALND in selected SLN-positive patients. This narrative review summarizes two decades of pivotal trial evidence across three conceptually distinct de-escalation strategies, which differ in their levels of evidence. First, phase III randomized evidence supports omission of ALND after a positive SLNB for isolated tumor cells and micrometastases (IBCSG 23-01; 10-year disease-free survival HR 0.85, with the non-inferiority criterion met), and for 1–2 macrometastases when combined with breast-conserving surgery and radiotherapy (ACOSOG Z0011, AMAROS, SENOMAC). Second, omission of SLNB itself has demonstrated 5-year oncological safety in rigorously selected low-risk patients with small tumors and negative preoperative axillary ultrasonography (SOUND, INSEMA), in cohorts that were predominantly HR-positive/HER2-negative. Third, emerging strategies, such as advanced imaging, ctDNA-based monitoring, and AI-assisted diagnostics, may further refine patient selection but remain investigational and are not part of established clinical practice at present. The current evidence is insufficient to support routine de-escalation in HER2-positive and triple-negative disease or in other populations underrepresented in the pivotal trials. Axillary de-escalation is therefore justified only within the specific clinical scenarios validated by randomized trials, and aggressive subtypes and biomarker-guided strategies require further prospective evidence before integration into clinical practice.

## 1 Introduction

Breast cancer is the prevalent malignancy among women globally, with more new cases diagnosed first, more of which are clinically node-negative (cN0) (1). Axillary lymph node status is a critical determinant of breast cancer staging and prognosis, with management strategies directly impacting treatment outcomes and quality of life (QoL) (2). Traditionally, axillary lymph node dissection (ALND) has been the gold standard for axillary evaluation and treatment. However, this procedure causes upper limb lymphedema (15%-30%), paresthesia (20%-40%), and limb dysfunction, which can severely impair long-term QoL (3, 4).

Currently, the core objective of axillary management in cN0 breast cancer is to minimize surgical trauma while ensuring tumor control (5). The sentinel lymph node (SLN), the primary node for lymphatic metastasis, provides the foundation for axillary de-escalation. Studies show that patients with SLN-negative status have <5% risk of subsequent nodal metastasis, thereby establishing SLN biopsy (SLNB) as the standard staging method for cN0 disease (6–8). Nevertheless, three core controversies remain: (1) How should optimal treatment be defined for different SLN metastatic burdens? (2) What are the precise selection criteria and eligible populations for axillary surgery omission? (3) Do safety boundaries for de-escalation differ across molecular subtypes? In this review, we have summarized the pivotal trials and recent evidence from the past two decades, and systematically analyzed the advances and controversies in cN0 axillary de-escalation to support individualized clinical decision-making. Most recently, randomized trials have challenged even the necessity of SLNB in selected patients, reflecting a broader shift from maximal intervention toward biologically optimized axillary management – a trajectory comprehensively explored in a recent evidence-based review spanning both upfront-surgery and post-neoadjuvant settings (9). Conceptually, axillary surgery de-escalation in cN0 breast cancer encompasses three distinct strategies that address different clinical questions and are supported by different levels of evidence: (1) omission of ALND after a positive SLNB, supported by phase III randomized trials (IBCSG 23-01, ACOSOG Z0011, AMAROS, and SENOMAC); (2) omission of SLNB itself in rigorously selected low-risk cN0 patients, as evaluated in the SOUND and INSEMA trials; and (3) emerging selection strategies based on imaging, molecular biomarkers, or circulating tumor DNA (ctDNA), which remain investigational. This review is organized accordingly, and evidence supporting one strategy should not be extrapolated to another. Terminology note: throughout this review, receptor status is designated as hormone receptor-positive/HER2-negative (HR+/HER2−) according to the definitions used in the cited trials, and the term “luminal” is reserved for the intrinsic (immunohistochemistry-based) subtype classification (Luminal A/Luminal B); survival endpoints — disease-free survival (DFS), recurrence-free survival (RFS), invasive disease-free survival (IDFS), and distant disease-free survival (DDFS) — are reported as defined in each original trial and are not interchangeable.

### 1.1 Literature search strategy

This is a narrative review. A literature search of PubMed/MEDLINE was performed (most recently updated in September 2026) for publications between January 2000 and December 2025, using combinations of the following search terms: (“breast cancer” OR “breast neoplasms”) AND (“sentinel lymph node” OR “sentinel node” OR “axillary lymph node dissection” OR “axillary dissection” OR “axillary surgery” OR “axillary de-escalation” OR “axillary radiotherapy” OR “regional nodal irradiation” OR “axillary management”).

The inclusion criteria were as follows: (1) phase III randomized controlled trials addressing axillary surgical management and oncological outcomes in clinically node-negative (cN0) breast cancer; (2) prospective cohort studies and large retrospective cohort studies reporting axillary surgical outcomes with adequate follow-up; (3) systematic reviews and meta-analyses on this topic; and (4) major national and international guidelines and staging manuals (e.g., NCCN guidelines, CACA guidelines, and the AJCC Cancer Staging Manual).

The exclusion criteria were as follows: (1) case reports and small single-center case series; (2) editorials, commentaries, and letters without original data; (3) studies confined exclusively to the neoadjuvant setting, which is beyond the scope of this review focusing on upfront-surgery cN0 disease; and (4) studies with duplicate or overlapping patient populations, for which the most recent or most comprehensive report was retained.

Titles and abstracts of the retrieved records were screened by the first author, and full texts of potentially relevant publications were reviewed against the eligibility criteria. In addition, seminal historical publications predating the search window (e.g., Saphir and Amromin, 1948) and key conference presentations of pivotal trials for which no full publication was available (e.g., the monarchE ctDNA analysis) were identified through backward citation tracking of the included articles and guideline documents. During the revision process, the search was updated, and additional recent publications identified through the reviewers’ suggestions were incorporated. The final selection prioritized evidence with direct clinical relevance to axillary de-escalation in cN0 breast cancer.

## 2 Strategy 1 — omission of ALND after positive SLNB: prognostic significance and management according to SLN metastatic burden

According to the AJCC TNM Staging Manual (8th Edition), SLN metastases are classified by burden: isolated tumor cells (ITCs; ≤0.2 mm maximum diameter or ≤200 cells per section), micrometastases (>0.2 mm to ≤2 mm or >200 cells), and macrometastases (>2 mm) (10). These categories exhibit distinct biological behaviors, prognostic relevance, and treatment strategies requiring individualized evidence-based decisions (**Table 1**). This section addresses the first de-escalation strategy: omission of ALND after a positive SLNB, stratified by SLN metastatic burden.

**Table 1:** Classification, prognosis, and management of SLN metastasis burden in cN0 breast cancer.

Category	Definition (AJCC 8th ed.)	Stage	Prognosis	Current management	Key evidence/guidelines
Isolated tumor cells (ITCs)	≤0.2 mm maximum diameter, or ≤200 cells per section	pN0(i+)	Locoregional recurrence risk comparable to pN0(i−) (5-year local recurrence 2.3% vs 1.9%, P = 0.22)	Treated as node-negative; ALND omission	Dutch national cohort (10); CACA/Chinese Society of Breast Surgery guidelines (ITC defined as SLN-negative, omission recommended, Grade 1A) (11)
Micrometastases	>0.2 mm to ≤2 mm, or >200 cells	pN1mi	Low burden; long-term outcomes unaffected by ALND omission	ALND omission for a single micrometastasis without high-risk features; axillary radiotherapy may be considered for multifocal micrometastases or high-risk factors after individualized assessment	IBCSG 23–01 (12); guidelines (11, 13, 14)
Macrometastases	>2 mm	pN1	Higher burden; management depends on nodal number and treatment context	1–2 macrometastases: ALND omission appropriate with BCS + whole-breast radiotherapy + systemic therapy (or axillary radiotherapy after mastectomy); ≥3 macrometastases, extensive LVI, or tumor >5 cm: ALND recommended	ACOSOG Z0011, AMAROS, SENOMAC (7, 15–18)

### 2.1 ITCs (pN0(i+))

ITCs were clinically first described in 1948 (12). According to the AJCC 6th Edition, breast cancer with detectable ITCs is staged as pN0(i+). Despite its non-impact on pathological staging, the presence of ITCs requires evidence-based optimization of management.

A Dutch retrospective study of 18,370 pT1-2N0-1miM0 patients demonstrated no significant difference in the 5-year local recurrence between pN0(i+) and pN0(i-) groups (2.3% vs. 1.9%, P = 0.22), and no elevated risk of distant metastasis (13). The IBCSG 23-01 10-year follow-up further showed that ALND omission in patients with low-burden sentinel-node metastases (≤2 mm, including ITCs) yielded comparable 10-year DFS to ALND (76.8% vs 74.9%; HR 0.85, 95% CI 0.65-1.11; log-rank P = 0.24; non-inferiority P = 0.0024), with no significant difference in overall survival (OS) between the two groups (11). These findings are consistent with a meta-analysis of 26 studies, which confirmed that omitting ALND in SLN-positive cN0 patients was not associated with increased mortality (OS: HR 0.97, 95% CI 0.92-1.03; P = 0.37) (15). Dutta et al. conducted a meta-analysis of 603 studies, and confirmed minimal DFS reduction with ALND omission (HR = 1.08, 95% CI: 0.92-1.27), with adjuvant therapy offsetting recurrence risk (19).

The current guidelines concur with these findings. For instance, the Chinese Society of Breast Surgery and CACA guidelines (2024) define ITC as SLN-negative, and recommend ALND omission (Grade IA evidence) (20). Accordingly, ITCs are managed as node-negative disease: neither completion ALND nor additional axillary radiotherapy is indicated on the basis of ITCs alone (10, 13, 20).

### 2.2 Micrometastases (pN1mi)

SLN micrometastasis detection rates have risen to 30%-40% among SLN-positive cases with improved early detection (21). Although the necessity of ALND was previously debated, multiple RCTs have validated the safety of ALND omission. IBCSG 23–01 was the first phase III trial that demonstrated the non-inferiority of ALND omission for SLN micrometastases. In a cohort of 934 patients with SLN micrometastasis, the 10-year DFS rates in the no-ALND (n=469) and ALND (n=465) groups were 76.8% (95% CI 72.5-81) and 74.9% (70.5-79.3) respectively (HR 0.85, 95% CI 0.65-1.11; log-rank P = 0.24; non-inferiority P = 0.0024) after median 9.7-year follow-up (IQR 7.8-12.7), which was consistent with the 5-year results and Z0011 10-year analysis (11, 22). These findings have also been corroborated by the Milan 185, ALMANAC, and NSABP B32 (6, 7, 14) trials, thereby establishing the evidence base for ALND omission in micrometastatic disease.

Current guidelines recommend ALND omission for cN0 patients with a single micrometastasis without high-risk features (i.e., tumor >2 cm, grade III, lymphovascular invasion) (16, 20). Multifocal micrometastasis or high-risk factors may warrant axillary radiotherapy after individualized benefit-risk assessment (19, 20).

### 2.3 Macrometastases (pN1)

The findings of the ACOSOG Z0011, AMAROS, and SENOMAC trials support axillary de-escalation for SLN macrometastases. The ACOSOG Z0011 study enrolled 891 cT1-2N0M0 patients with 1–2 metastatic SLNs, all of whom underwent breast-conserving surgery followed by tangential whole-breast radiotherapy. At a median follow-up of 9.3 years, 10-year OS was 86.3% with SLNB alone versus 83.6% with ALND (HR 0.85; one-sided 95% CI 0-1.16; non-inferiority P = 0.02), and 10-year DFS was 80.2% versus 78.2% respectively (HR 0.85; 95% CI 0.62-1.17; P = 0.32), with no significant difference in regional recurrence (17, 18). Surgical morbidity, including lymphedema, was significantly lower with SLNB alone than with ALND (23). These results challenged the consensus of mandatory ALND for limited SLN metastasis. Notably, the Z0011 conclusions are restricted to the population and treatment context evaluated — cT1–2 tumors, 1–2 positive sentinel nodes, breast conservation with whole-breast radiotherapy, and systemic therapy — and the presence of 1–2 positive sentinel lymph nodes should not, by itself, be regarded as sufficient justification for ALND omission in all clinical settings (e.g., after mastectomy or without adjuvant radiotherapy). Accordingly, NCCN guidelines recommend ALND omission only for T1-2, breast-conserved, radiotherapy-treated patients with 1–2 SLN macrometastases (category 2A) (16).

AMAROS further expanded the eligibility for ALND omission; a total of 1425 cT1-2N0 SLN-positive patients (60.4% macrometastases, 861/1425) were enrolled, with 76.7% presenting single-node and 18.3% presenting two-node involvement. The patients who underwent mastectomy (18%) were randomized to the ALND and axillary radiotherapy arms. The 10-year axillary recurrence rate after ALND was 0.93% (95% CI 0.18-1.68; 7 events) compared to 1.82% (95% CI 0.74-2.94; 11 events) in the axillary radiotherapy group (HR 1.71; 95% CI 0.67-4.39) (24). The OS (HR 1.17; 95% CI 0.89-1.52) and DFS (HR 1.19; 95% CI 0.97-1.46) rates were comparable, but surgical morbidity was significantly lower with radiotherapy (11.9% vs. 24.5%, P<0.001) (25). The wide 95% confidence intervals around the 10-year axillary recurrence rates reflect the very small number of events (7 vs 11), which limited the statistical power of the non-inferiority comparison. The trial was designed with a non-inferiority margin of 2% for 5-year axillary recurrence (assuming 2% in the ALND group and accepting up to 4% in the radiotherapy group), and the planned non-inferiority test was already underpowered at the 5-year analysis because the observed event rates were far lower than anticipated (25). Consequently, formal non-inferiority cannot be statistically established, and a small absolute detriment of axillary radiotherapy cannot be excluded. Nevertheless, when interpreted in absolute terms, the 10-year axillary recurrence rates were below 2% in both arms; even the upper bounds of the 95% confidence intervals (1.68% and 2.94% respectively) remained below the 4% threshold pre-specified as acceptable for the radiotherapy group, and the absolute difference was only 0.89 percentage points. Along with the absence of any difference in OS, DFS, and locoregional control, and with significantly less arm morbidity after radiotherapy (11.9% vs 24.5%, P<0.001), these data support axillary radiotherapy as a reasonable alternative to ALND for SLN-positive cT1-2N0 patients — particularly those undergoing mastectomy or those intolerant to ALND — while acknowledging the residual statistical uncertainty (24, 25).

SENOMAC, the largest phase III trial addressing SLN macrometastases, enrolled 2766 cT1-3N0 patients with 1–2 SLN macrometastases across five countries (2540 in the per-protocol population), randomizing them into completion ALND versus SLNB only. Reflecting a broader clinical context than Z0011, the trial included tumors up to cT3 and patients undergoing mastectomy, and approximately 89% of patients in both arms received radiotherapy including nodal target volumes; median follow-up was 46.8 months. Five-year RFS was 89.7% with SLNB only versus 88.7% with completion ALND (country-adjusted HR 0.89; 95% CI 0.66-1.19). As this confidence interval includes 1, the result does not demonstrate a significant inter-group difference; the reported P<0.001 refers to the prespecified non-inferiority analysis, indicating that the hazard ratio was significantly below the non-inferiority margin. Subgroup analyses showed consistent results across breast-conserving and mastectomy settings, providing the strongest evidence to date for ALND omission in patients with 1–2 SLN macrometastases, although the relatively short median follow-up warrants cautious interpretation (26). A recent meta-analysis of 15 studies (six RCTs and nine cohort studies), encompassing 33,599 patients treated with SLNB alone and 95,711 receiving ALND, further confirmed that omitting ALND in cT1–2 cN0 patients with 1–2 SLN macrometastases does not compromise DFS (HR = 0.99, 95% CI: 0.85-1.14, P = 0.857) or OS (HR = 1.03, 95% CI: 0.92-1.14, P = 0.251) (27).

Current consensus indicates that ALND omission is appropriate for cN0 patients with 1–2 SLN macrometastases who meet the following criteria: (1) breast-conserving surgery + whole-breast radiotherapy; (2) no significant LVI; (3) appropriate systemic therapy — with explicit consideration of anticipated treatment adherence, given that the safety of omission demonstrated in pivotal trials was contingent on high rates of adjuvant systemic therapy (28, 29). Mastectomy patients may consider axillary radiotherapy as an ALND alternative, contingent on tumor size and molecular subtype (16, 25); consistent with this, the 2025 ASCO guideline update supports omitting ALND in patients with cN0 tumors ≤5 cm and one to two positive sentinel nodes who undergo mastectomy, provided postmastectomy radiotherapy with regional nodal irradiation is delivered (30). For patients with ≥3 SLN macrometastases, extensive lymphovascular invasion, or tumor size >5 cm, ALND is recommended to ensure locoregional control (16, 20). The pivotal randomized trials underpinning axillary surgery de-escalation in cN0 breast cancer are summarized in **Table 2**.

**Table 2:** Pivotal randomized trials of axillary surgery de-escalation in cN0 breast cancer.

Trial (reference)	Population	Comparison	Surgery/radiotherapy context	Primary endpoint	Median follow-up	Main result
IBCSG 23-01 (12, 20)	cN0 (non-palpable nodes), tumor ≤5 cm, SLN micrometastases (≤2 mm), no extracapsular extension; n=934	ALND vs no ALND	BCS or mastectomy; RT per institutional practice	DFS	9.7 years (10-year update)	10-year DFS 74.9% (ALND) vs 76.8% (no ALND); HR 0.85 (95% CI 0.65–1.11); log-rank P = 0.24 (no significant difference); prespecified non-inferiority P = 0.0024
ACOSOG Z0011 (21, 22)	cT1-2N0, 1–2 positive SLNs; n=891	ALND vs SLNB alone	BCS + tangential whole-breast RT mandatory (*post-hoc* analysis: 81.1% tangents only, 18.9% directed nodal RT)	OS	9.3 years (10-year update)	10-year OS 83.6% (ALND) vs 86.3% (SLNB); HR 0.85 (one-sided 95% CI 0–1.16); non-inferiority P = 0.02; 10-year DFS 78.2% vs 80.2%, HR 0.85 (95% CI 0.62–1.17), P = 0.32 (no significant difference)
AMAROS (15, 28)	cT1-2N0, positive SLN (60.4% macrometastases); n=1425	ALND vs axillary RT	BCS (82%) or mastectomy (18%); comparison arm received axillary RT	Axillary recurrence rate	6.1 years (5-year analysis); 10-year update	5-year ARR 0.43% (ALND) vs 1.19% (ART); planned non-inferiority test underpowered; 10-year ARR 0.93% vs 1.82% (HR 1.71, 95% CI 0.67–4.39); lymphedema 24.5% vs 11.9% (P<0.001, favoring ART)
SENOMAC (18)	cT1-3N0, 1–2 SLN macrometastases; n=2540 (per protocol)	Completion ALND vs SLNB only	BCS and mastectomy; ~89% in both arms received nodal RT	RFS	46.8 months	5-year RFS 88.7% (ALND) vs 89.7% (SLNB only); HR 0.89 (95% CI 0.66–1.19); P<0.001 for the prespecified non-inferiority margin (not a superiority test)
SOUND (24)	cN0, tumor ≤2 cm, negative axillary ultrasonography; n=1405	SLNB vs no axillary surgery	BCS + whole-breast RT mandatory	Distant disease-free survival (DDFS)	5.7 years	5-year DDFS 97.7% (SLNB) vs 98.0% (no surgery); HR 0.84 (90% CI 0.45–1.54); log-rank P = 0.67 (no significant difference); non-inferiority P = 0.02
INSEMA (25)	cT1–2 cN0 (clinical and imaging assessment); n=4858 (per protocol)	SLNB vs omission of axillary surgery	BCS + whole-breast RT mandatory	Invasive DFS (iDFS)	73.6 months	5-year iDFS 91.7% (SLNB) vs 91.9% (omission); HR 0.91 (95% CI 0.73–1.14), below the prespecified non-inferiority margin; axillary recurrence (first event) 0.3% vs 1.0%

ALND, axillary lymph node dissection; SLNB, sentinel lymph node biopsy; BCS, breast-conserving surgery; RT, radiotherapy; ART, axillary radiotherapy; DFS, disease-free survival; OS, overall survival; RFS, recurrence-free survival; DDFS, distant disease-free survival; iDFS, invasive disease-free survival; ARR, axillary recurrence rate; HR, hazard ratio; CI, confidence interval.

## 3 Strategies 2 and 3 — omission of SLNB in selected cN0 patients and emerging selection approaches

While SLNB has reduced unnecessary ALND, it remains invasive and may lead to minor complications such as hematoma and sensory abnormalities in 5%-10% of the patients (3). Advances in imaging and systemic therapy efficacy have shifted focus toward direct axillary surgery omission (including SLNB) for selected low-risk cN0 patients. The core principle is the precise identification of populations with ultra-low recurrence risk through non-invasive or minimally invasive means to avoid surgical trauma. This section addresses a strategy distinct from that discussed in Section 1: omission of SLNB itself in rigorously selected cN0 patients, as evaluated in the SOUND and INSEMA trials, rather than omission of ALND after a positive SLNB. The selection approaches discussed in Sections 2.2 and 2.3 (molecular features, imaging, and ctDNA) represent a third strategy that remains investigational.

### 3.1 Multimodal imaging-guided selection

Preoperative axillary ultrasound is the standard approach for cN0 assessment, with biopsy specificity of 100% and sensitivity of 79.6% to exclude occult positive nodes (31). The SOUND trial was the first phase III study to explore the feasibility of SLNB omission in cN0 patients, but its findings apply to a strictly selected population: women with tumors ≤2 cm, negative preoperative axillary ultrasonography, and undergoing breast-conserving surgery and whole-breast radiotherapy with guideline-directed adjuvant therapy (median age 60 years; median tumor size 1.1 cm). Notably, 87.8% of participants had ER-positive/HER2-negative tumors, although eligibility was not restricted to this subtype. At a median follow-up of 5.7 years, the primary endpoint — 5-year distant DFS — was 97.7% in the SLNB group versus 98% in the no-axillary-surgery group (HR 0.84; 90% CI 0.45-1.54); the conventional inter-group comparison showed no significant difference (log-rank P = 0.67), and the pre-specified non-inferiority criterion was met (non-inferiority P = 0.02) (32). Notably, the trial did not identify any subgroup — defined by age, tumor size, histologic grade, Ki-67 index, or molecular subtype — in which SLNB omission was associated with a higher risk of recurrence; however, no formal subgroup analyses were performed because of the very low number of events (axillary recurrence as a first event in 0.4% vs 0.7% of patients), and higher-risk subgroups were underrepresented in the enrolled population (patients younger than 40 years, 1.4%; grade 3 tumors, approximately 18%; HER2-positive disease, 6.8%; triple-negative disease, 5.4%), precluding definitive conclusions regarding the safety of omission in these subgroups (32). The INSEMA trial subsequently confirmed these findings in a broader but still imaging-selected population of patients with cT1–2 cN0 tumors undergoing breast conservation and whole-breast radiotherapy: at a median follow-up of 73.6 months, 5-year invasive DFS was 91.9% with omission versus 91.7% with SLNB (HR 0.91; 95% CI 0.73-1.14), meeting the pre-specified non-inferiority criterion, although axillary recurrence as a first event was numerically more frequent with omission (1.0% vs 0.3%) (33). Together, these trials support SLNB omission only in rigorously imaging-selected, low-risk patients treated with breast conservation and whole-breast radiotherapy, and should not be interpreted as evidence for routine SLNB omission in all cN0 patients (32, 33) (**Table 2**). Consistent with this, the 2025 ASCO guideline update recommends against routine SLNB in selected postmenopausal patients aged ≥50 years with grade 1-2, small (≤2 cm), HR-positive/HER2-negative tumors and negative preoperative axillary ultrasound who undergo breast-conserving therapy (30). However, a 2026 systematic review and meta-analysis of five studies (8108 patients) found that omission of axillary surgery was associated with a higher risk of axillary recurrence (RR 3.82, 95% CI 1.48-9.82, P<0.005), despite no significant differences in DFS (RR 1.09, 95% CI 0.91-1.30) or OS (RR 1.06, 95% CI 0.72-1.55), thus underscoring that omission should be restricted to highly selected patients with favorable tumor biology, and that multidisciplinary input remains essential for adjuvant treatment planning when nodal staging is omitted (34).

Emerging modalities, including axillary MRI and PET-CT, may further refine cN0 assessment: MRI provides clearer visualization of nodal morphology and extracapsular extension, while PET-CT assesses metastatic risk through metabolic activity, particularly for larger tumors or high-risk subtypes (20, 31). However, these modalities are supported mainly by retrospective or single-center diagnostic studies, and no randomized trial has established imaging-based selection criteria beyond the axillary-ultrasonography-based selection validated in SOUND and INSEMA. Multimodal imaging strategies should therefore be regarded as adjunctive tools that complement — rather than replace — the selection approach validated in prospective trials (31–33).

### 3.2 Integration of molecular and clinical features

The molecular subtype and clinicopathological features of breast tumors also provide key information for patient selection. For instance, HR-positive/HER2-negative tumors generally exhibit more indolent biology; in the SOUND and INSEMA cohorts — which were predominantly HR-positive/HER2-negative — 5-year axillary recurrence rates after SLNB omission were only 0.7%-1% (32, 33). Conversely, triple negative breast cancer (TNBC) and HER2+ breast cancer, despite neoadjuvant sensitivity, are underrepresented in de-escalation trials; therefore, the current evidence is insufficient for routine SLNB omission (16, 20).

Clinically, age, tumor size, and histological grade are key prognostic factors. The strongest guideline endorsement for SLNB omission concerns older patients: the Society of Surgical Oncology’s Choosing Wisely recommendation advises against routine SLNB in clinically node-negative women aged ≥70 years with early-stage, HR-positive/HER2-negative tumors (35), and NCCN guidelines likewise support omission of axillary staging in this setting when nodal status would not alter adjuvant treatment decisions (16). Randomized evidence in women aged ≥70 years with small, ER-positive, clinically node-negative tumors further showed that de-escalation of locoregional therapy did not compromise long-term survival (36). However, chronological age alone should not determine eligibility; life expectancy, comorbidities, tumor biology, and whether nodal status would change systemic therapy should all be integrated into the decision (16, 37). Consistent with this, a meta-analysis of 44 real-world studies in patients aged ≥65 years found that omitting SLNB or ALND did not significantly reduce overall survival (37). Younger patients (<40 years) with tumors >3cm, grade III staging, or LVI have a higher risk of axillary metastasis, thereby warranting cautious evaluation (16, 20).

### 3.3 Liquid biopsy as an ancillary selection tool for axillary de-escalation

Liquid biopsy-based detection of ctDNA has emerged as a promising tool for prognostic assessment in breast cancer (38). In the monarchE trial — which enrolled patients with HR-positive, HER2-negative, node-positive, high-risk early breast cancer receiving adjuvant abemaciclib combined with endocrine therapy (39, 40) — ctDNA was successfully detected in the plasma samples of 910 patients (65% of 1397) through whole-exome sequencing. The IDFS event rate was 87% in ctDNA-positive patients compared with only 15% in persistently ctDNA-negative patients across all evaluated timepoints (pre-treatment, and 3, 6, and 24 months post-treatment). Patients who achieved ctDNA clearance during treatment had a lower risk of IDFS events than those who remained ctDNA-positive, although the event risk remained clinically meaningful even after clearance (39). These data were reported in abstract form at the 2024 ASCO Annual Meeting; full peer-reviewed publication is pending, and the figures should be interpreted accordingly. Likewise, a *post-hoc* analysis of the SENOMAC trial addressed whether completion ALND is warranted solely to identify pN2–3 status as an indication for adjuvant CDK4/6 inhibitor therapy: an estimated 104 completion ALNDs would be required to avoid one 5-year invasive DFS event with adjuvant abemaciclib, at the cost of nine patients experiencing severe or very severe arm morbidity, arguing against ALND performed purely for systemic-therapy selection (41). These data establish ctDNA dynamics as a strong prognostic marker for recurrence risk in the adjuvant setting.

However, it must be emphasized that the monarchE population — high-risk, node-positive patients treated with adjuvant systemic therapy — is fundamentally different from the cN0 population that is the focus of this review, and these data were generated for prognostic monitoring rather than for primary surgical decision-making. Extrapolating ctDNA clearance data from a high-risk adjuvant setting to justify surgical omission in a low-risk cN0 setting is not currently supported by any prospective surgical trial. Therefore, although preoperative ctDNA negativity in cN0 patients is biologically plausible as an indicator of minimal disease burden, ctDNA assessment should at present be regarded strictly as a prognostic monitoring tool; its use in guiding the omission of axillary surgery remains investigational and requires dedicated prospective validation, with predefined detection thresholds and demonstrated incremental value beyond multimodal imaging and clinicopathological features (38). Future integration of multimodal imaging, molecular subtype, clinicopathological features, and ctDNA holds promise for further reducing the false-negative rate of axillary surgery omission, but such strategies must be validated in prospective trials before clinical implementation.

### 3.4 Implementation requirements and follow-up strategies

Current axillary omission strategies require: (1) strict selection criteria; (2) comprehensive systemic therapy (endocrine or targeted) — with explicit assessment of anticipated adherence, since early discontinuation of adjuvant endocrine therapy (approximately 31% of patients) and suboptimal adherence (28% of continuers) are common and independently associated with increased mortality (HR 1.26, 95% CI 1.09-1.46; and HR 1.49, 95% CI 1.23-1.81, respectively) (28), and adherence below 80% is associated with poorer survival (HR 1.20, 95% CI 1.03-1.40; P = 0.019) (29); as patients omitting axillary staging may harbor occult residual nodal disease whose control relies on systemic therapy, premature discontinuation could erode the safety margin demonstrated in clinical trials, and intensified surveillance should be considered in such cases; (3) structured follow-up consistent with current guidelines, comprising clinical examination and axillary ultrasonography, with the surveillance schedules used in the pivotal omission trials serving as a pragmatic reference (32, 33); serial ctDNA monitoring is not an established surveillance strategy, and its role in follow-up after axillary omission remains investigational and should be confined to clinical studies (38). Suspicious nodes (cortical thickening, loss of normal architecture) warrant prompt biopsy and potential ALND or axillary radiotherapy supplementation. Intensive follow-up increases the chances of detecting delayed axillary recurrence, and salvage therapy can achieve favorable OS, thus highlighting its critical role in implementing safe omission.

## 4 Molecular subtype subgroup analysis: the key aspect of precision de-escalation

Different molecular subtypes exhibit distinct biological behaviors and tolerability to de-escalation.

HR-positive/HER2-negative (luminal-like) breast cancer comprises 60%-70% of cN0 cases; however, this group is biologically heterogeneous: Luminal A tumors (high ER/PR expression, low Ki-67) typically exhibit indolent biology, low recurrence risk, and sensitivity to endocrine therapy, whereas Luminal B (HER2-negative) tumors frequently harbor high Ki-67 indices and display more aggressive clinical behavior and a higher recurrence risk (42, 43). The ACOSOG Z0011 trial reported no significant difference in 10-year DFS between SLNB alone and ALND in the overall trial population — which was predominantly ER-positive — (80.2% vs 78.2%; HR 0.85, 95% CI 0.62-1.17; P = 0.32), with only one regional recurrence in the SLNB-alone group between years 5 and 10, and no significant difference in 10-year regional recurrence (17). However, outcomes were not reported separately for Luminal A versus Luminal B subtypes. In the SENOMAC trial, approximately 85% of the patients had ER+/HER2- tumors with 1–2 SLN macrometastases (median follow-up 46.8 months). Five-year RFS was 89.7% (95% CI: 87.5-91.9) with SLNB alone compared to 88.7% (95% CI: 86.3-91.1) with ALND, with a nation-adjusted recurrence/death HR of 0.89 (95% CI: 0.66-1.19) that was well below the non-inferiority margin (P<0.001) (26). Taken together, no pivotal de-escalation trial has reported outcomes stratified by Luminal A versus Luminal B (HER2-negative) disease; therefore, current evidence supports axillary de-escalation primarily for ER-positive/HER2-negative tumors with low proliferative activity, whereas patients with Luminal B tumors and high Ki-67 indices should be evaluated individually — with integration of proliferative markers and genomic risk assessment — pending subtype-stratified prospective data (42, 43).

Patients with ER-positive/HER2-negative tumors constituted the majority of the populations enrolled in the SLNB-omission trials (e.g., 87.8% in SOUND); therefore, the safety of omission is best established in this group (32, 33). Nevertheless, trial eligibility criteria should not be relaxed on the basis of subtype alone: SOUND and INSEMA combined molecular features with strict imaging and clinical selection (small tumors, negative axillary ultrasonography, breast conservation with whole-breast radiotherapy) (32, 33). Luminal biology should thus be viewed as one favorable component of a multifactorial selection strategy, rather than as a stand-alone criterion for omission.

Multiple trials (SOUND, INSEMA) have explored the oncological safety of SLNB omission. However, HER2+ and TNBC patients comprised <10% of the cohort, resulting in insufficient evidence for de-escalation in these high-risk subtypes. Notably, any apparent safety signal in these subgroups derives from trials in which HER2-positive and triple-negative patients were markedly underrepresented; it should therefore be regarded as hypothesis-generating rather than practice-changing. At present, SLNB remains the standard of care for these subtypes, and omission of axillary surgery should be offered only within prospective clinical trials.

### 4.1 Discussion and controversies

#### 4.1.1 Safety boundary controversies in de-escalation

The safety evidence reviewed above derives from two distinct clinical scenarios — omission of ALND after a positive SLNB (IBCSG 23-01, ACOSOG Z0011, AMAROS, SENOMAC) and omission of SLNB in selected patients (SOUND, INSEMA) — and these two bodies of evidence are not interchangeable. The central issue in cN0 axillary de-escalation is defining the safety boundaries. While ACOSOG Z0011 and SENOMAC confirm the safety of ALND omission for 1–2 SLN macrometastases, there is a lack of consensus for tumors >3 cm, LVI, or high-risk subtypes (e.g., TNBC) (17, 26). Some researchers argue that the high risk of occult axillary metastasis makes ALND omission unsafe, while others contend that systemic therapy and radiotherapy adequately control local disease, with limited ALND benefit and significant morbidity (23, 25).

Additionally, the metastasis number cutoff is controversial. It should be emphasized that the “1-2” threshold adopted by current guidelines is primarily empirical rather than biological: it reflects the eligibility criteria of the pivotal trials (ACOSOG Z0011, AMAROS, and SENOMAC), all of which enrolled patients with 1–2 positive sentinel nodes (17, 25, 26). The oncological rationale for caution beyond this threshold is that the number of involved sentinel nodes serves as a surrogate for total axillary tumor burden: as the number of positive sentinel nodes increases, the probability of residual non-sentinel nodal metastases rises — 27% of patients in the ALND arm of Z0011 harbored additional positive nodes (44) — and the risks of locoregional failure and systemic dissemination increase accordingly (45). Physiologically, because axillary lymphatic drainage is sequential, a greater number of positive sentinel nodes implies a higher likelihood that metastatic disease has extended beyond the sentinel basin into higher axillary echelons that are not encompassed by tangential breast irradiation. Nevertheless, no sharp biological boundary exists between 2 and 3 positive nodes — the AJCC staging system itself groups 1–3 positive nodes as pN1 (10) — and some data show no significant difference in recurrence for three micrometastases (19). Therefore, the absolute number of affected nodes should not be regarded as an absolute limit; rather, total metastatic burden, extracapsular extension, and molecular subtype should be integrated into multidimensional risk-stratification models to define more precise safety boundaries (45). The clinical scenarios in which each de-escalation strategy is currently supported are summarized in **Table 3**.

**Table 3:** Clinical scenarios and currently supported axillary de-escalation strategies in cN0 breast cancer.

Clinical scenario	Currently supported strategy	Level of supporting evidence
cN0, SLNB negative	No further axillary surgery	Established (phase III trials, guidelines)
cN0, SLNB with isolated tumor cells	ALND omission; manage as node-negative	Established (guidelines, cohort data)
cN0, SLNB with a single micrometastasis, no high-risk features	ALND omission	Established (IBCSG 23-01)
cN0, 1–2 SLN macrometastases, undergoing BCS + whole-breast RT + systemic therapy	ALND omission	Established (ACOSOG Z0011; SENOMAC)
cN0, 1–2 SLN macrometastases, undergoing mastectomy	Axillary radiotherapy as an alternative to ALND; individualized decision	Supported (AMAROS; SENOMAC subgroup), with caveats regarding statistical power
cN0, ≥3 positive SLNs, extensive LVI, or tumor >5 cm	ALND recommended	Established (guidelines)
Selected low-risk cN0 (small tumor, negative axillary imaging, favorable biology, BCS + whole-breast RT; e.g., postmenopausal HR-positive/HER2-negative)	SLNB omission may be considered in rigorously selected patients	Supported by SOUND and INSEMA; reflected in the 2025 ASCO guideline update
High-risk biological subtypes (triple-negative, HER2-positive)	SLNB remains standard; omission only within clinical trials	Limited; investigational
Biomarker- or ctDNA-guided omission of axillary staging	Not recommended outside research settings	Investigational; prospective validation required

#### 4.1.2 Role of radiotherapy in de-escalation

The role of radiotherapy in de-escalation also requires clarification. Although the ACOSOG Z0011 (17) and SOUND (32) trials have been criticized for heterogeneity in radiotherapy delivery, the radiation fields used in Z0011 have been explicitly characterized in a dedicated secondary analysis. Jagsi et al. retrospectively reviewed the radiation records of trial participants and found that among 605 evaluable patients, 89% received whole-breast irradiation and 15% received additional supraclavicular treatment; among the 228 patients with detailed records available for central review, 81.1% were treated with tangential fields alone, high tangents (cranial border ≤2 cm from the humeral head, thereby incidentally encompassing axillary levels I–II) were used in 50% of patients in the ALND arm and 52.6% in the SLNB arm, and 18.9% (43/228) received directed regional nodal irradiation with three or more fields, with no significant difference between the arms in the use of protocol-prohibited nodal fields (46). These data indicate that the oncological safety of ALND omission in Z0011 was achieved predominantly with tangential whole-breast irradiation — frequently with high tangents — rather than with comprehensive regional nodal irradiation. Similarly, in the INSEMA (33) and BOOG 2013–08 trials, radiotherapy was delivered to axillary level I in >25% and 1.5% of patients respectively, despite SLNB omission.

In contrast, the AMAROS trial confirmed axillary radiotherapy as an alternative to ALND for SLN-positive patients, with fields encompassing the full axilla (levels I–III) plus the supraclavicular fossa (24, 25). However, whether such comprehensive regional nodal irradiation (RNI) truly represents de-escalation warrants critical appraisal. Although the 10-year AMAROS analysis demonstrated durable axillary control with significantly less lymphedema than ALND (11.9% vs 24.5% at 5 years, P<0.001) (24), RNI substitutes one set of toxicities for another: in the MA.20 trial, adding RNI to whole-breast irradiation significantly increased grade ≥2 acute pneumonitis (1.2% vs 0.2%, P = 0.01) and lymphedema (8.4% vs 4.5%, P = 0.001) (47), and an exploratory AMAROS analysis suggested a numerically higher 10-year incidence of second primary cancers after axillary radiotherapy (12.1% vs 8.3%) (24). RNI should therefore be regarded as a substitution of morbidities — trading the surgical complications of ALND for radiation-associated toxicities — rather than a morbidity-free de-escalation. By contrast, the high-tangent fields used in most Z0011 patients provide partial axillary coverage (levels I–II) with less added toxicity and may be sufficient for Z0011-eligible patients, whereas full RNI, despite being a reasonable alternative for patients undergoing mastectomy or those in whom ALND is not feasible, should be reserved for patients in whom the anticipated benefit justifies the additional radiation toxicity.

For micrometastases or ITCs, the necessity and field remain controversial; there is evidence that minimal local recurrence risk obviates benefit, while potentially increasing cardiovascular toxicity and skin fibrosis (11, 19). Nevertheless, some questions persist: 1) Does radiotherapy merely maintain standard fields to enable surgery omission, or must dose/fields be intensified to replace surgery? 2) What tumor burden thresholds justify radiotherapy substitution? 3) Can surgical de-escalation be independent of radiotherapy?

#### 4.1.3 Standardization of pathological assessment

Accurate SLN burden assessment depends on standardized protocols, yet practices vary across centers, resulting in discrepancies in diagnostic performance (20). Major international pathology guidelines provide specific recommendations. The College of American Pathologists (CAP) protocol recommends that each sentinel node be grossly sliced at intervals of no more than 2 mm perpendicular to its long axis, that all slices be entirely submitted for histological examination, and that evaluation be based on hematoxylin-eosin (H&E) stained sections, with routine cytokeratin immunohistochemistry not required for H&E-negative nodes (48), which is supported by randomized evidence showing that occult sentinel-node metastases detected only by immunohistochemistry have no clinically meaningful impact on survival (49). The European Working Group for Breast Screening Pathology similarly recommends gross slicing at approximately 2-mm intervals combined with step sectioning (multiple H&E levels through the tissue block), with selective cytokeratin immunohistochemistry (e.g., AE1/AE3) reserved for morphologically equivocal foci and for lobular carcinoma rather than routine use (50, 51). It should be noted that these sectioning and staining recommendations derive from professional pathology society guidelines and expert consensus (48, 50, 51) rather than from randomized trial evidence, and therefore represent accepted best-practice standards for sentinel node assessment. The direct impact of these parameters on the safety of axillary de-escalation has not been tested prospectively. Notably, “sectioning thickness” comprises three distinct parameters that are often conflated: the gross slicing interval (≤2 mm), the microtome section thickness (typically 3-5 μm), and the interval between step levels; genuine standardization requires specifying all three. Regardless of the detection method (H&E or immunohistochemistry), nodal deposits are classified strictly by size according to the AJCC 8th edition as ITCs (≤0.2 mm or ≤200 cells), micrometastases (>0.2–2 mm), or macrometastases (>2 mm) (10). Nevertheless, ambiguous lesions (e.g., measuring 0.20-0.30 mm) can still be classified as ITCs or micrometastases depending on the center, thereby impacting treatment decisions (10). Establishing standardized SLN pathology protocols is therefore critical for precise de-escalation. Digital pathology and AI-assisted image analysis are emerging tools that may improve detection reproducibility in the future; however, current evidence derives largely from retrospective validation studies, and these approaches remain investigational and are not yet incorporated into pathology guidelines for sentinel node assessment (48, 50).

## 5 Conclusions and future directions

Current evidence supports axillary surgery de-escalation in cN0 breast cancer only within specific, well-defined clinical scenarios, each resting on a distinct evidence base. First, omission of ALND after a positive SLNB is supported by phase III randomized evidence for isolated tumor cells and micrometastases (IBCSG 23-01) and for 1–2 macrometastases in patients treated with breast-conserving surgery plus whole-breast radiotherapy (ACOSOG Z0011, AMAROS, SENOMAC). Second, omission of SLNB itself is supported by randomized evidence (SOUND, INSEMA) only in rigorously selected low-risk patients — those with small tumors, negative preoperative axillary ultrasonography, predominantly HR-positive/HER2-negative disease, and guideline-directed adjuvant therapy. Third, emerging selection strategies based on advanced imaging, ctDNA, or AI-assisted diagnostics remain investigational and should not yet influence surgical decision-making outside clinical trials. Accordingly, de-escalation should not be extrapolated beyond these evidence-based boundaries: axillary staging with standard surgical management remains the reference standard in biologically more aggressive subtypes (HER2-positive and triple-negative disease), in younger patients, and in clinical presentations outside the eligibility criteria of pivotal trials. Future research priorities include: (1) prospective validation of the long-term safety of omission strategies in populations underrepresented in the pivotal trials, particularly HER2-positive and triple-negative disease, and younger patients; (2) prospective evaluation of biomarker-guided selection, including ctDNA and AI-assisted diagnostics, before any role in surgical decision-making can be considered; (3) establishment of internationally standardized sentinel-node pathology protocols and definition of the optimal surveillance strategy after omission; (4) clarification of the role of radiotherapy in omission strategies; and (5) real-world studies on applicability, systemic-therapy adherence, and cost-effectiveness. Until such evidence is available, axillary de-escalation in cN0 breast cancer should be implemented only within the selection criteria validated by the pivotal trials — moving from a paradigm of maximum tolerable treatment toward one of minimum effective therapy, applied only when supported by trial-level evidence. Through these efforts, we can expand the applicability of axillary de-escalation in cN0 breast cancer, thereby achieving the goal of precision therapy, minimal trauma, and optimal outcomes, while providing high-level evidence to update clinical guidelines and improve patient QoL and long-term prognosis.
